# Non-targeted analysis (NTA) and suspect screening analysis (SSA): a review of examining the chemical exposome

**DOI:** 10.1038/s41370-023-00574-6

**Published:** 2023-06-28

**Authors:** Katherine E. Manz, Anna Feerick, Joseph M. Braun, Yong-Lai Feng, Amber Hall, Jeremy Koelmel, Carlos Manzano, Seth R. Newton, Kurt D. Pennell, Benjamin J. Place, Krystal J. Godri Pollitt, Carsten Prasse, Joshua A. Young

**Affiliations:** 1 School of Engineering, Brown University, Providence, RI 02912 USA; 2 Agricultural & Environmental Chemistry Graduate Group, University of California, Davis, Davis, CA 95616 USA; 3 Department of Epidemiology, Brown University, Providence, RI 02912 USA; 4 Exposure and Biomonitoring Division, Environmental Health Science and Research Bureau, Health Canada, Ottawa, ON Canada; 5 Department of Environmental Health Sciences, Yale School of Public Health, New Haven, CT 06520 USA; 6 Department of Chemistry, Faculty of Science, University of Chile, Santiago, RM Chile; 7 School of Public Health, San Diego State University, San Diego, CA USA; 8 Office of Research and Development, U.S. Environmental Protection Agency, Washington, DC USA; 9 National Institute of Standards and Technology, 100 Bureau Dr, Gaithersburg, MD 20899 USA; 10 Department of Environmental Health & Engineering, Johns Hopkins University, Baltimore, MD 21205 USA; 11 Risk Sciences and Public Policy Institute, Bloomberg School of Public Health, Johns Hopkins University, Baltimore, MD 21205 USA; 12 Division of Biology, Chemistry and Materials Science, Office of Science and Engineering Laboratories, Center for Devices and Radiological Health, Food and Drug Administration, Silver Spring, MD 20993 USA

**Keywords:** Non-targeted analysis, Suspect screening analysis, High-resolution mass spectrometry, Exposome, Environmental media, Chemical space

## Abstract

**Abstract:**

Non-targeted analysis (NTA) and suspect screening analysis (SSA) are powerful techniques that rely on high-resolution mass spectrometry (HRMS) and computational tools to detect and identify unknown or suspected chemicals in the exposome. Fully understanding the chemical exposome requires characterization of both environmental media and human specimens. As such, we conducted a review to examine the use of different NTA and SSA methods in various exposure media and human samples, including the results and chemicals detected. The literature review was conducted by searching literature databases, such as PubMed and Web of Science, for keywords, such as “non-targeted analysis”, “suspect screening analysis” and the exposure media. Sources of human exposure to environmental chemicals discussed in this review include water, air, soil/sediment, dust, and food and consumer products. The use of NTA for exposure discovery in human biospecimen is also reviewed. The chemical space that has been captured using NTA varies by media analyzed and analytical platform. In each media the chemicals that were frequently detected using NTA were: per- and polyfluoroalkyl substances (PFAS) and pharmaceuticals in water, pesticides and polyaromatic hydrocarbons (PAHs) in soil and sediment, volatile and semi-volatile organic compounds in air, flame retardants in dust, plasticizers in consumer products, and plasticizers, pesticides, and halogenated compounds in human samples. Some studies reviewed herein used both liquid chromatography (LC) and gas chromatography (GC) HRMS to increase the detected chemical space (16%); however, the majority (51%) only used LC-HRMS and fewer used GC-HRMS (32%). Finally, we identify knowledge and technology gaps that must be overcome to fully assess potential chemical exposures using NTA. Understanding the chemical space is essential to identifying and prioritizing gaps in our understanding of exposure sources and prior exposures.

**Impact statement:**

This review examines the results and chemicals detected by analyzing exposure media and human samples using high-resolution mass spectrometry based non-targeted analysis (NTA) and suspect screening analysis (SSA).

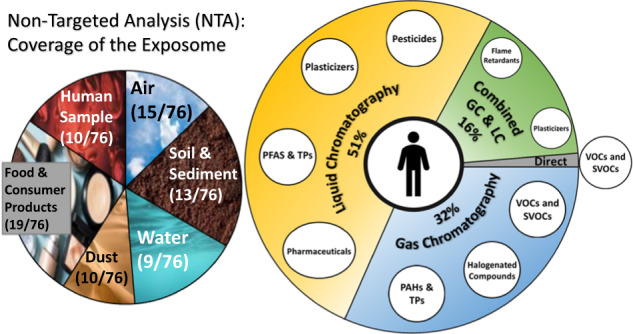

## Introduction

Human exposure to environmental chemicals is ubiquitous given the widespread use of chemicals in consumer products, food, manufacturing, building materials, furnishings, and industrial processes. Anthropogenic chemicals and their transformation products can adversely influence health and represent a major contributor to the exposome, or the totality of exposures individuals experience during their life [1, 2]. While there are several approaches to measuring the chemical exposome, high-resolution mass spectrometry (HRMS) coupled with liquid or gas chromatography (LC or GC) has enabled researchers to successfully develop and employ non-targeted analysis (NTA) into their experimental repertoire. NTA is a discovery-based approach for detection of organic chemicals that does not require a priori knowledge of the species present in the sample. Examples of NTA include suspect screening analysis (SSA), as defined by the Benchmarking and Publications for Non-Targeted Analysis (BP4NTA) Working Group and unknown compound analysis [3]. In SSA, molecular features are compared against databases containing chemical suspects to identify potential matches [4]. In true NTA, unknown compounds are postulated without suspect lists. Since SSA is a subcategory of NTA, we will use the term NTA in the remainder of this manuscript to refer to the use of both true NTA and SSA approaches. NTA has allowed for the development of analytical workflows that can characterize human exposures in multiple environmental media (air, water, dust, soil) and human samples beyond the pollutants traditionally assessed in targeted analysis, which typically focus on a relatively small number (e.g., <100) of chemical species. Although NTA has provided researchers the opportunity to explore a greater portion of the chemical exposome, the use of GC or LC based methods does not cover all the potential chemical components. For example, larger chemical species (>1500 Da) and metals require different analytical approaches.

Understanding and properly accounting for the vast diversity of chemicals which may be detectable (or not detectable) in a sample is one of the most important and difficult aspects of designing a NTA study to assess chemical exposures. The “chemical space” may be thought of as a conceptual yet exhaustive collection of all possible chemicals that exist within a sample [5]. While some study objectives attempt to evaluate as much of the chemical space as possible, other NTA studies have been intentionally focused on evaluation of a particular region of the chemical subspace, such as looking for nonspecific polycyclic aromatic hydrocarbons (PAH). The study objectives and subspace of organic chemicals relevant to a study are thus important factors for understanding and choosing analytical conditions used in an NTA study because the conditions will dictate which chemicals are found [6]. As reported by Black et al., eight key analytical considerations heavily influence the detected chemicals space: (1) sample matrix type, (2) extraction solvent, (3) pH, (4) extraction/cleanup media, (5) elution buffers, (6) instrument platform, (7) ionization type, and (8) ionization mode [7]. Several approaches can be used to help visualize the chemical space from physicochemical properties (such as LogP, molecular weight, octanol-air partition coefficient, or soil adsorption coefficient). The final subspace of chemicals detectable after accounting for all analytical considerations (such as extraction solvents and pH, the use of solid phase extraction, elution buffers, chromatography conditions, and mass spectral ionization type and mode) has been termed the “detectable space” and is critical in assessing the appropriateness of the selected analytical conditions for the intended application [7]. For example, the chromatography chosen, whether gas or liquid, will influence the detectable space, which has been previously discussed by Zhang et al. [8]. LC is more amenable to water soluble compounds with polar functional groups that ionize under atmospheric pressure; however, GC is more amenable to more non-polar, volatile compounds [8].

The objective of this paper is to review the published literature reporting the use of NTA for assessing sources of chemical exposure from environmental media, the chemical space generally observed in the media, and the assessment of exposures in humans. To accomplish this objective, this manuscript discusses the value of NTA for measuring the chemical exposome, methods and challenges of using NTA to characterize chemical exposures in environmental matrices and human samples, the current gaps in knowledge and technology for applying NTA in exposure assessment studies, and the implications and benefits of using NTA in public health research. In doing so, we will elucidate the chemical space that has already been captured in environmental and human samples, the analytical techniques that have been used to identify these chemical exposures, and identify the gaps in chemicals detected in each sample media.

## Value of NTA for exposome characterization

Humans are exposed to a complex range of chemicals and their transformation products in their daily lives. Environmental exposures occur through “aggregate exposure” (exposure to a single known chemical by a single or multiple exposure routes and from different sources) or through “cumulative exposure” (exposure to a chemical by a single or multiple sources over an extended period of time) [9]. Humans may be exposed to a single chemical or multiple chemicals simultaneously. Exposure research is typically hypothesis driven; for example, researchers suspect that a known volatile or semi volatile organic compound (VOC or SVOC) is linked to a health outcome and will only analyze for the particular VOC stated in their hypothesis. However, adverse health effects could be due to other unknown or unsuspected chemicals that co-occur with that VOC or the combined effects of multiple VOCs. NTA is a discovery-based analytical approach to detect organic chemicals unsuspected a priori; providing more comprehensive exposure assessments and potentially leading to the identification of single or combinations of multiple chemicals that are associated with adverse health outcomes.

## Characterization of exposure sources using NTA

**Fig. 1:**
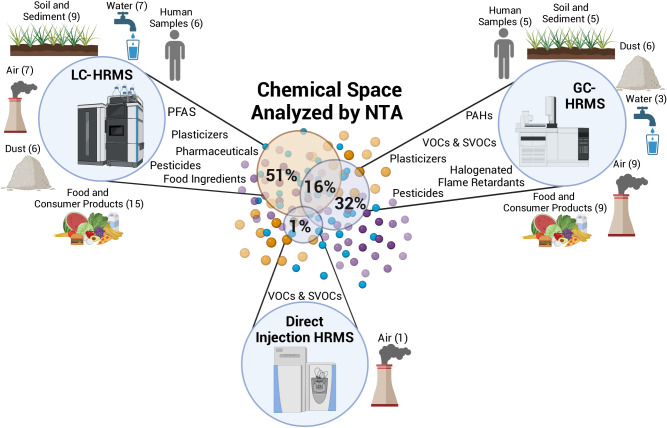
Schematic diagram of the chemical space analyzed by HRMS using NTA. The number and percentage of studies using liquid chromatography (LC), gas chromatography (GC), or direct injection paired with high-resolution mass spectrometry (HRMS) are shown, including the types of chemicals that were detected and the media that have been characterized using each approach. Figure created with BioRender.com.

The results obtained for measurements of the chemical exposome using HRMS based NTA varies depending on the media, extraction method, and HRMS analytical platform used. Below, we reviewed the methods and challenges associated with extracting and measuring organic chemicals in various media, including water, soil and sediment, air, dust, food and consumer products (such as medical devices and tobacco products), as well as human samples, for exposure assessment using NTA. In total, 76 manuscripts were considered in this review (Fig. 1). The review was conducted using the Abstract Sifter tool [10] to search PubMed and using the Web of Science, and by searching for keywords, such as “non-targeted analysis”, “suspect screening analysis” and the exposure media.

**Fig. 2:**
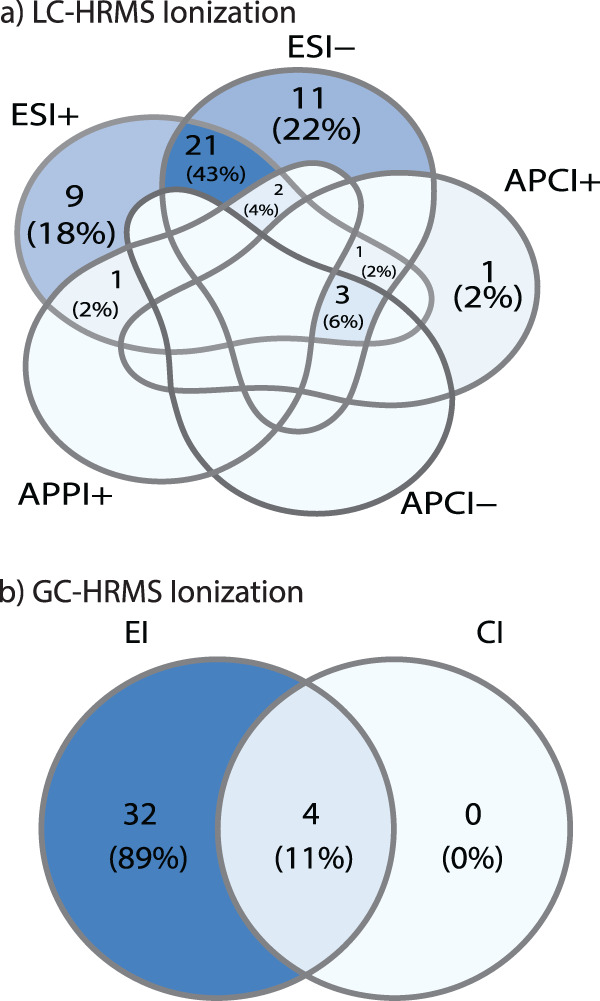
A summary of the ionization types used in the LC- and GC-HRMS NTA studies reviewed. The type of ionization used in liquid chromatography (electrospray (ESI) in positive or negative mode, atmospheric pressure chemical ionization (APCI) in positive or negative mode, or atmospheric pressure photoionization (APPI)) is in (**a**). The type of ionization used in GC (electron ionization (EI) or chemical ionization (CI)) is displayed in (**b**). Unlabeled intersections in the Venn Diagram had zero frequency.

Important and impactful research has been conducted on the characterization of exposures using NTA; however, the number of published studies and research progress made has not been equal across analytical platforms, chemical classes, and environmental matrices. Overall, 51% of the studies considered herein used only LC-HRMS, 32% used only GC-HRMS, 16% used both GC-HRMS and LC-HRMS, and 1% used a direct injection approach (no chromatography was used and the sample was directly injected onto the mass spectrometer) (Fig. 1). Of the papers that used LC-HRMS, many (43%) used both negative and positive electrospray ionization (ESI− and ESI+); however, some only used one ESI ionization mode (18% ESI+, 22% ESI−) (Fig. 2a). Other ionization techniques (i.e., atmospheric pressure chemical ionization (APCI)) were used less often. In papers that used GC-HRMS, all used electron ionization (EI), and occasionally complemented the EI with chemical ionization (CI) (11% of the papers) (Fig. 2b).

Furthermore, many of the studies considered herein used SSA and less used true NTA. The most common approach for putatively identifying compounds in GC-HRMS was searching the NIST Mass Spectral Library, which contains unit-mass resolution mass spectra. Out of the GC-HRMS papers reviewed, only two used true NTA approaches [11, 12]. In the first study by Zhang et al., an in silico approach was used to isolate halogenated compounds in dust [11]. Similarly, the objective of the second study was to detect halogenated compounds in water samples; thus, the NTA approach used Kendrick mass defect plots to first isolate halogenated compounds, then used isotope distributions to determine the number of bromide and chloride atoms in the molecule, and then confirmed elemental compositions with theoretical isotope distributions [12]. In the LC-HRMS studies reviewed, 21 papers used SSA, 15 used true NTA, and 15 used both approaches. In many studies using both NTA and SSA, Compound Discoverer (CD), a commercially available software by Thermo, was commonly used to search mass spectra against databases (mass lists, in-house MS^2^ databases, mzVault, and mzCloud) for SSA or to generate molecular formulas using CD’s Predicted Composition node for NTA. When computational tools used to analyze the data were specified, most studies (*n* = 57) used vendor software, such as Thermo Compound Discoverer or Agilent MassHunter, to perform their analysis. Only 7 studies included in this review used open-source software, which included MzMine, TracMass, and MS-DIAL [13–19]. One study combined open-source software (for peak integration) with vendor software (for identification) [20]. One study used custom workflow that was not open source [21]. Thus, a gap in current NTA/SSA research is the availability of open-source software that employs true NTA for both GC and LC HRMS platforms.

### Water

Humans are exposed to chemicals in water through ingestion and dermal contact. While few NTA studies have focused on drinking water samples, the majority of water-focused NTA studies have focused on wastewater [22–24] and surface or ground water [25–27]. The dearth of studies performing NTA directly on drinking water may be attributed to higher contaminant concentrations in surface water and wastewater compared to drinking water. One strategy to address sensitivity has been to preconcentrate contaminants on point-of-use filters during drinking water sampling [28]. Using this strategy, NTA revealed that the drinking water tested contained Per- and Polyfluoroalkyl Substances (PFAS), chlorinated phosphate esters, pesticides, antimicrobials, food additives, personal care products, and chemicals used in industrial processes.

Wastewater provides an efficient means to surveil community levels of synthetic chemicals, including pharmaceuticals, drugs of abuse, and emerging organic contaminants [24, 29]. Many emerging organic contaminants originate in consumer products and enter waste streams via discharge from residential drainage [11]. This provides insight regarding product usage, including unexpected compound classes such as triclosan metabolites, halogenated anisoles, and phthalates [11, 30]. Triclosan is an antibacterial used in many consumer products, including soaps and detergents and skin care products. Similarly, phthalates are present in many cosmetics and are also used in plastics as plasticizers; they are considered a group of priority substances by the European Union [30]. Anisoles are listed as Persistent Organic Pollutants (POPs) under the Stockholm Convention and are either used in or are transformation products of pesticides and flame retardants [11]. Thus, sources of chemicals in wastewater reflect products used by humans in their everyday lives and monitoring wastewater using NTA could provide useful information about what is being discharged and influence treatment design and decisions.

A common approach in NTA water monitoring studies is to focus on a class of compounds, such as PFAS. Often this limited focus is defined at the outset of the study, but in other cases researchers allow preliminary data analysis to direct their focus. Ostensibly, this does not fit into the naïve (or “hypothesis free”) nature of an NTA approach and can limit a study’s findings. However, in practice, there is no NTA method that can screen for all organic chemicals in a single sample due to the diversity of physicochemical properties in environmental chemicals. Therefore, these studies can still be considered NTA as they do not target specific compounds, only compound classes, and focus on novel compound discovery which is a major goal of NTA. For example, focusing on unknown HRMS features that are indicative of fluorinated organic compounds (using the characteristically low-to-negative mass defect of the compounds) allowed for the discovery of a novel and important PFAS compound in surface waters that recharge local wells in New Jersey [25]. Another study focused on antimicrobial agents and used in silico tools to predict transformation products of these agents, which could then be screened for in the HRMS data. In these cases, the use of strategic data reduction strategies aided the discovery of novel compounds and transformation products, respectively.

#### Chemicals detected

**Fig. 3:**
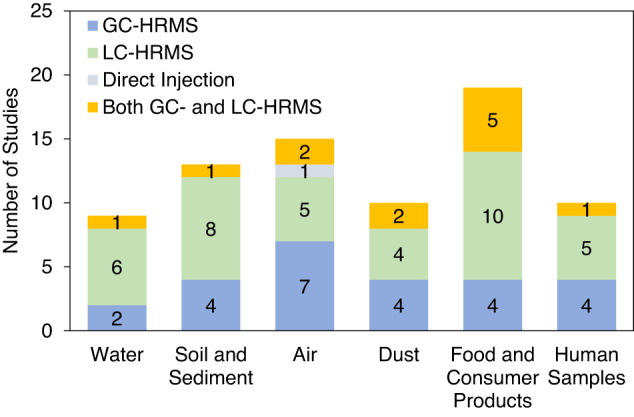
A Summary of the HRMS technique used for NTA in each environmental media reviewed. The chromatography technique (GC, LC, and/or Direct Injection) chosen in the HRMS NTA studies for each environmental media reviewed, including water, soil and sediment, air, dust, food and consumer products, and human samples.

Of the NTA studies on water considered in this review on the NTA, six used LC-HRMS, two used GC-HRMS, and one used both GC- and LC-HRMS (Fig. 3). NTA studies of drinking water typically use LC instrumentation as this platform is generally well suited for the analysis of relatively low volatility, water-soluble compounds. LC-HRMS based NTA has identified pharmaceuticals, PFAS, pesticides, plasticizers, halogenated and non-halogenated flame retardants, and transformation products of these chemical classes in water. On the Environmental Protection Agency (EPA) CompTox Chemicals Dashboard, a tool that can be utilized to curate mass lists for SSA, there are 44 lists containing 36,640 chemicals detectable in water [31]. Of these lists, only one is specific to GC-HRMS amenable compounds and includes 814 chemicals, including disinfection byproducts (DBP), pesticides, and PAHs. In order to use mass lists in GC-HRMS, it is important to consider ionization and fragmentation for the desired chemical space. For example, soft ionization techniques, such as chemical ionization, would be required to observe the identifiable molecular ion peak; however soft ionization may not be efficient. In the characterization of water, GC-HRMS has identified DBPs formed in water treatment and aliphatic compounds, organic acids, and saturated and unsaturated hydrocarbons and oxygenated compounds in hydraulic fracturing wastewaters [32, 33].

### Soil and sediment

Humans are exposed to chemicals that accumulate in soil and sediment through non-dietary ingestion (e.g., pica behavior), inhalation (e.g., vapor intrusion), and dermal contact (e.g., recreational activities such as wading, fishing, or swimming). NTA studies on soil and sediment have contributed to our understanding of these media as an exposure source [34, 35]. Lipophilic chemicals, which have high n-octanol-water partition coefficient (*K*_OW_) values, accumulate in soil and sediment due to accidental spills, agricultural applications, water reuse, and biosolids application (among other causes).

NTA of soils has been performed on a wide range of locations ranging from as large as across a single country (Switzerland) to specific locations with unique exposures such as fire training facilities, coke manufacturing plants, and agricultural settings [13, 34–36]. Chiaia-Hernández et al. examined soil and sediment samples collected across the country of Switzerland using LC-HRMS NTA [34]. The study demonstrated that soil and sediment contain a different set of environmental chemicals, as only 34 of the 96 compounds identified were detected in both matrices. Furthermore, soils collected near agriculture and urban settings and sediments impacted by agriculture and wastewater treatment plants had the highest detection rates of organic chemicals. In agricultural settings, high detection of organic chemicals is concerning considering the potential uptake into edible plants and crops. NTA has revealed that crops contain a number of chemicals, including human and veterinary pharmaceuticals, due to the application of organic waste products to the field (i.e., animal waste, reclaimed water) [35, 37].

In locations with manufacturing and waste facilities, NTA has been used to better understand industrial discharges that may result in human exposure. For example, sediment sampled from a river that receives discharge from a fluoropolymer manufacturing plant in China analyzed using LC-HRMS NTA revealed that the river (which provides well water and crop irrigation) contained PFAS without well-documented uses [38]. A possible source of these not well documented PFAS is biotic transformation, which has been examined in sediment using NTA in laboratory-scale experiments [39]. Aside from PFAS, extracts from sediment sampled near an e-waste recycling plant analyzed using both GC- and LC-HRMS revealed that the sediment contained PAHs and PAH transformation products, including hetero-PAHs, n-hetero-PAHs, and di-/ter-phenyls [40]. In location specific studies, NTA can also be useful to find novel emerging persistent organic pollutants. Liquid crystal monomers are an example of a new chemical class that has been found in soil, sediment, and outdoor dust around e-waste recycling facilities [41].

#### Chemicals detected

In the papers reviewed on sediment and soils, eight papers used LC-HRMS, four papers used GC-HRMS and one paper used both LC- and GC-HRMS (Fig. 3). NTA has revealed that soil and sediment accumulate a range of chemicals and transformation products, including PFAS, pesticides, herbicides, synthetic musks, steroids, pharmaceuticals, food additives, polychlorinated biphenyls (PCBs), organophosphate esters, liquid crystal monomers, PAHs, and alkyl-PAHs (PAH transformation products) [13, 21, 34–36, 40–45]. Much of the current NTA research on soil and sediment is based on SSA; thus, NTA focused on unknown compounds is necessary to explore the full chemical profile that may be present in soils.

### Air

Humans are exposed to environmental chemicals in indoor and outdoor air through inhalation. Outdoor air pollutants can enter indoor spaces through openings or cracks in buildings and through engineered ventilation. Poor ventilation/filtration can lead to elevated concentrations of airborne contaminants indoors, where humans spend the majority of their time. Items in the built environment, such as building materials, furnishings, fuel-burning appliances, personal care products, and other consumer products also release airborne contaminants through off-gassing or direct use and release.

NTA has been extensively used to study the chemical composition of gas and particle-phase chemicals in samples collected using active and passive methods [14, 15, 46–49]. NTA has also been used to evaluate the potential distribution between air and water matrices in the environment [49]. For example, Chung et al. paired NTA with fugacity models, which are chemical models describing equilibria between environmental phases, and found that diethylene glycol monoethyl ether was detectable in air and water samples, but nine chemicals were only detected in air and 14 were only detected in water [49]. Most of these studies have evaluated temporal variation of these environmental chemicals across the day or between seasons. For example, Giorio et al. found that a group of aromatic and poly-aromatic compounds increased during winter months in Italy [14]. Future studies should consider employing NTA for examining the spatial variation of environmental chemicals.

Since the distribution of airborne environmental chemicals is heterogenous due to different local emission sources and the potential for long-range transport phenomena, NTA has been used to study source specific emissions, such as e-waste recycling facilities [50], woodsmoke emissions [51], and the effect of removal processes such as rain or snowfall [52]. In smoke from a fire in a storage site for recycled resources, halogenated compounds and dioxins were detected [50]. In locations with oil reserves, specifically where petroleum coke (“petcoke,” a potential energy source) is stockpiled outdoors, heterocyclic aromatics and N-containing heterocyclic PACs (NPACs) with alkylated carbazoles, benzocarbazoles, and indenoquinolines isomer groups were detected in outdoor air [52].

NTA has been used to evaluate the composition of airborne environmental chemicals in real-world and simulated indoor environments [53, 54]. Method development studies focusing on resolving the chromatograms of complex mixtures in indoor environments [55] have helped to increase the number of annotated chemical features in indoor environments by NTA, specifically using SSA. This type of research could improve the estimation of other important air-sampling parameters, such as the cotton-air partition coefficient [56], and help to understand the contributions of the indoor environment to the chemical exposome. Several studies have explored personal exposure to airborne chemicals. These studies commonly use wearable passive samplers and can represent a combination of indoor and outdoor exposures [57]. The exposures include chemicals linked to insecticides, diet, personal care products, cigarette smoke, sunscreen, antimicrobial soaps, energy production (PAHs), and aromatics. NTA has revealed that placement of personal exposure devices (i.e., whether the device is on a person’s chest, wrist, or shoe) alters the chemical space detected for indoor and outdoor exposures [58]. In this study, VOCs were less impacted by sampler body placement; while shoe placement had increased levels of particle-bound SVOCs [59].

#### Chemicals detected

The chemical space covered in air related studies using NTA is varied, particularly because of the use of different techniques for sampling, processing, and analysis. There is a growing tendency to include novel sample collection techniques in NTA studies focused on air, including cryogenic air sampling [46], sampling with a combination of sorbent tubes [59], and passive sampling [49, 52, 57]. In the studies reviewed pertaining to air, five studies only used LC-HRMS, seven studies only used GC-HRMS, two studies used both LC-HRMS and GC-HRMS, and one study used a direct injection approach (Fig. 2). The types of compounds that have been identified in the indoor environment include terpenoids, alkaloids, triglycerides, sterol derived chemicals, carotenoids, fatty acids, cotinine, nicotine, furfural, petrochemicals, PFAS, phthalates, flame retardants, pesticides, flavoring agents, fragrances, terpenes, straight chain alkanes, cycloalkanes, and polycyclic aromatic compounds [53–55, 58, 59]. The types of compounds that have been detected in urban outdoor air include alkanes, cycloalkanes, levoglucosan, aldehydes, ketones, amines, sulfates and sulfonates, carboxylic acids, benzenes, benzoic acids, phenols (including nonylphenol), nitrophenols, polycyclic aromatic compounds (parental, nitrogen, sulfur, and oxygen containing heterocycles), phthalates, organophosphates, organosulfates, and PFAS [14, 15, 46–52, 57].

### Dust

Dust is known to be a sink for many household environmental chemicals [20]. Humans can be exposed to chemicals in dust through inhalation, ingestion, and dermal pathways. Over 200 chemicals have been found in the National Institute of Standards and Technology (NIST) Standard Reference Materials (SRM) 2585 Organic Contaminants in House Dust; the list is available on EPA’s CompTox Chemicals Dashboard and can be exported as a mass list for use in NTA, specifically in SSA (https://comptox.epa.gov/dashboard/chemical-lists/NISTSRM2585) [20, 31]. Thus, dust has been comprehensively characterized using both GC- and LC-based NTA in workflows that were not specific to certain compound classes. Only two studies considered in this review focused their NTA efforts on a specific compound class or group (OPEs and halogenated compounds), rather than taking a generalized approach [12, 60]. Rostowski et. al. used dust as a matrix for assessing intra-laboratory variability of NTA and found that of the 2350 compounds (18% of these were identification confidence level 1, 25% were level 2, and 58% were level 3 on the Schymanski scale [61]) that they were able to identify in dust, 5% were detected by both LC- and GC-HRMS [62]. In this NTA study, dust samples were analyzed by 21 laboratories and 41% of the compounds reported could not be detected by more than one laboratory. Dust has been used for semi-quantitative NTA [20, 63], which was used in turn to estimate the potential toxicity of exposure in toddlers. Semi-quantitative NTA approaches provide an estimated concentration for the detected exposures by correcting the peak area of the discovered chemical using another chemical or an internal standard with a known concentration. This approach is used to help prioritize detected exposures; however, this approach does have limitations, which are discussed below. Personal exposure studies that assess dust could help prioritize chemicals for biomonitoring, develop studies for assessment of health risk concerns, and be used to develop exposure mitigation strategies.

#### Chemicals detected

Both LC- and GC-HRMS platforms have been used to characterize indoor dust. In most cases, one analytical platform is chosen; however, there are a few studies that characterize settled dust using both platforms. Four studies reviewed in this paper characterized dust using only LC-HRMS, four studies used only GC-HRMS, and two studies used both LC-HRMS and GC-HRMS (Fig. 3). Using LC-HRMS, NTA has revealed that indoor dust contains PFAS, parabens, and nicotine [20, 64, 65]. GC-HRMS has revealed that indoor dust contains organophosphates dyes, PAHs, and halogenated compounds [16, 66]. Using both LC- and GC-HRMS increased the chemical space of the studies and enabled a more comprehensive analysis of dust [67]. The chemicals detected using both analytical platforms included fatty acids, polyethylene glycols, plastics additives, including phthalates, organophosphate esters, flavor and fragrances, polycyclic aromatic hydrocarbons, pesticides, illicit drugs, and chemicals commonly found in personal care products.

### Food and consumer products

Consumer products can be a source of anthropogenic environmental chemicals. Here, we consider the environmental chemicals present in food, medical devices, and other commercial products (such as personal care products). When determining exposure to humans, the chemical can be an intended component of the product (e.g., plasticizer in some medical devices) or an unintended component of the product (e.g., pesticides in food). Using NTA of these products directly, the potential contribution of identified environmental chemicals to the human exposome can be evaluated.

#### Food

Contamination of food can occur through many routes, from the food production phase and through the food processing phase [17]. The primary exposure route to environmental chemicals from food is through ingestion but can also occur through inhalation or dermal contact as revealed by NTA of personal air samplers [58]. The environmental chemicals that have been identified in food products (including dietary supplements) by NTA can often be separated between chemicals intentionally or unintentionally used in food production or processing. These chemical species may not be intentionally added to food itself, such as pesticides [18, 68]. Chemicals that unintentionally enter the food stream include pharmaceuticals and ingredients in personal care products [69, 70]. Commercial-use chemicals, such as herbicides and PFAS [71, 72], have also been detected in food via NTA. In addition to the food itself, researchers have studied materials and packaging in contact with food as a potential source of environmental chemicals [73] and have shown the potential for possible leaching of contaminants into food [74–76].

#### Medical devices

Exposure to chemicals from medical devices can be through a variety of routes, including inhalation, ingestion, dermal absorption and direct exposure from internal implantation. To address these potential exposures, NTA is frequently utilized in the evaluation of plastics used in healthcare applications as part of biocompatibility assessment prior to clinical application [77]. In this context, the final product is exposed to a series of solvents encompassing varying polarities and any chemicals extracted are evaluated to determine the potential for any of the extractables to elicit toxicological harm if a patient is exposed to them [78]. Some of these extracted chemicals have been classified as environmental chemicals, and therefore are relevant to determining the total human exposure. Several authors have provided reviews of NTA practices for identifying extractable and leachable compounds and have attempted to use them to identify extractable chemicals of concern in commonly used medical materials [77, 79–81].

As the implementation of NTA in these applications has increased, organizations such as the Product Quality Research Institute (PQRI) arose to improve reliability and demonstrate best practices in such studies, specifically for orally inhaled nasal drug products [82]. For medical devices, a recent and comprehensive perspective on the use of NTA in chemical characterization of medical devices in regulatory submissions presented the relevant concepts of using NTA for evaluation of medical devices [83]. The utility of NTA is currently expanding beyond preclinical evaluation of these materials where evaluation is conducted from hypothetical “worst-case” solvents and extraction conditions. For example, NTA was recently used for the evaluation of volatile organic compounds (VOCs) in human milk when exposed to an enteral feeding system. The results demonstrated cyclohexanone and 3,3,5-trimethylcyclohexanone (compounds associated with the manufacture of the feeding system) accumulated significantly in milk samples post-infusion [84].

#### Other consumer products

Environmental chemicals have been identified using NTA in other unclassified consumer products such as stationary [85], clothing [86], personal care products [87], and tobacco products [88]. Phillips et al. utilized a broad sampling scheme to identify environmental chemicals in a wide variety of consumer products [4]. PFAS have been identified by NTA in cosmetics and other personal care products. Harris et al. reported a relationship between detected PFAS and intentional use of fluoropolymer in cosmetics [87].

#### Chemicals detected in consumer products

The chemical space covered using NTA in consumer products is varied, which relates to the broad properties and exposure routes of materials and chemicals in the consumer product categorization. In the papers considered for this review, ten studies used LC-HRMS, four studies used GC-HRMS, and five studies used both GC- and LC-HRMS (Fig. 3). For NTA of food products, instrumental techniques include LC-HRMS and GC-MS, as dependent on the intended detectable space and suspect chemical properties. Pesticides can be detected using LC-MS and GC-MS, but other compounds, such as PFAS and pharmaceuticals, with low vapor pressures tend to be more easily detected using LC-MS.

The chemical constituents on or in a medical device may come from disparate sources and encompass a wide range of chemical functionality. As a result, the detectable space of sample is vast and significantly influenced by extraction process. To address this, selection of extraction solvents and temperature is often based on guidance from published relevant standards, such as ISO (“the International Organization for Standardization”) 10993–18 [77]. For other consumer products, such as cosmetics or children’s clothing, the sample processing and techniques used for NTA can be more variable. In Bentley et al., the researchers used GCxGC-TOF-MS and LC-HRMS (with multiple ionization modes) to increase the breadth of their chemical space to achieve “full characterization” of the chemical composition of a heated tobacco product [88].

### Human samples

A range of human bodily fluids and tissues have been characterized using NTA to assess exposure through the measurement of parent compounds or their metabolites. By examining human specimens, we can better determine which environmental chemicals enter the human body and their potential health-related risks. Thus, a few occupational studies with NTA have been performed to help define the exposures.

LC-HRMS is the dominant analytical technique used for characterizing the exposome in human samples using NTA due to its use in metabolomics [89]. However, GC-HRMS is also gaining popularity for characterizing human samples with NTA because of the coverage of environmental chemicals. Using LC-HRMS, PFAS and cyclic volatile methylsiloxanes were identified using NTA of cord blood and placenta [90]. Use of GC-HRMS NTA has enabled detection of halogenated compounds, non-halogenated cyclic and aromatic compounds, pesticides, brominated flame retardants, PCBs, PAHs, phthalates, dyes, and plasticizers in human plasma, amniotic fluid, and breastmilk [91–93]. EPA’s CompTox Chemicals Dashboard [31] was used to categorize chemicals detected by NTA using GC-HRMS in plasma from 75 50-year-old people from Uppsala, Sweden [93]. The categorization revealed that chemicals from lists associated with human health risks (androgen or estrogen receptor activity, neurotoxin or potential neurotoxin, endocrine disruptor, and skin sensitizer) were associated with chemicals from lists describing consumer products (rubber, plastics, and cosmetics). Very few studies have conducted analyses of human samples using both LC- and GC-HRMS. Pourchet et al. used both LC (in negative and positive electrospray ionization) and GC-HRMS to develop an approach for characterizing breastmilk using NTA [19]. This study focused on non-polar environmental chemicals; GC-HRMS detected the widest range of non-polar chemicals (log p octanol/water: 2.2–8), followed by LC-HRMS negative ionization (log p octanol/water: 3.1–7.2), and LC-HRMS positive ionization covered the smallest polarity range (log p octanol/water: 2.2–4.6). Thus, polarity is an important consideration in choosing instrumentation and defining the detectable space of environmental exposures in human samples.

In addition to measurement of parent compounds, NTA has been used to detect metabolites, or biomarkers, of these exposure compounds. These biomarkers are not the exposure compounds, but their phase I or II metabolites. A recent study applied NTA to explore biomarkers of exposure in the urine of 200 children from 6 to 9 years old from Slovenia and identified 74 biomarkers at Schymanski confidence levels of 2 and 3 [61], including transformation products of pharmaceuticals, personal care products, plasticizers and plastic related products, volatile organic compounds, nicotine, caffeine and pesticides [94]. NTA has also been used to examine chemical-class specific metabolites, with a focus on phthalates. For example, in a study by Guo et al., LC-HRMS was used to identify direct metabolites of phthalate exposure in urine [95]. Nine known phthalate metabolites were detected, and a novel metabolite was identified (1,2-benzenedicarboxylic acid, mono(9-cyclopropylnonyl) ester).

While several studies assessing exposure to specific environmental chemicals or groups of chemicals exist, the literature to date on the use of NTA on human samples for the assessment of occupational exposures is still limited. Existing research has assessed exposures in firefighters, with a focus on PFAS [96–98]. Wallace et al. [96] used a NTA workflow for the analysis of breath samples from firefighters using low resolution GC-MS with the aim of identifying exposures. Exhaled breath samples were collected before, immediately after, and 1 h after entering a controlled burn. For structural elucidation of the detected features, mass spectra were compared to NIST library. In total 60 features were identified of which 7 were significantly more abundant (based on peak area) in post- vs. pre-firefighting samples. Even though no attempts to identify the compounds were made, the detected features were used to elucidate individuals showing highest exposures.

Rotander et al. [98] investigated exposures to known and unknown PFAS originating from aqueous film forming foam (AFFF) in firefighters using a combined targeted and non-targeted LC-HRMS workflow. In total 20 blood serum samples from firefighters and 19 from university students and office workers, who had not been exposed to AFFF were used. Identification of detected features was performed based on MS and MS/MS information by comparison with databases searches. Comparison of AFFF from different manufacturers were further used to identify potential PFAS sources in the firefighters. The results demonstrated that serum from firefighters contained several PFAS that were not included in the target list.

Grashow et al. [97] investigated chemical exposures in 83 women firefighters and 79 women office workers using negative ionization LC-HRMS analysis of serum samples. Detected features were matched with a custom chemical database of 722 slightly polar phenolic and acidic compounds, including many of relevance to firefighting or breast cancer etiology. Among the 620 chemicals that were detected, 300 had matching molecular formulas in the database, including phthalate metabolites, phosphate flame-retardant metabolites, phenols, pesticides, nitro and nitroso compounds, and PFAS. Among the detected compounds, 20 were selected for confirmation using reference standards and 8 were confirmed including two alkylphenols, ethyl paraben, bisphenol F, perfluorooctane sulfonamidoacetate, benzophenone-3, benzyl p-hydroxybenzoate, and triphenyl phosphate.

Newmeyer et al. investigated the chemical exposures in urine from 23 US-based female Black and Latina hairdressers serving an ethnically diverse clientele and 17 female office workers as the control group [99]. Using a LC-HRMS SSA approach, 24 compounds with median peak areas ≥2× greater among hairdressers compared to office workers were detected, including methylparaben, ethylparaben, propylparaben, and 2-naphthol. Categorization of the chemicals revealed most were associated with “personal use,” including 11 different “hair styling and care” products and fragrances, hair and skin conditioning, hair dyeing, and UV stabilizer compounds.

#### Chemicals detected

In human samples, both environmental chemicals and their metabolites have been detected using NTA. In the reviewed papers, ten studies used LC-HRMS, four studies used GC-HRMS, and five studies used both LC- and GC-HRMS to characterize exposures in human samples (Fig. 3). Using LC-HRMS, the chemicals detected included halogenated organics, phthalates and phthalate metabolites, PFAS, and hair products ingredients. Using GC-HRMS, volatiles, aromatics, aldehydes, alkanes, alkenes, phthalates, and halogenated compounds were detected. For human samples, the sample matrix is important when considering the detectable space and choosing the appropriate analytical method. For example, urine has high water content and is likely to contain hydrophilic (chemicals with low *K*_ow_ values) environmental chemicals; whereas fat tissue is likely to accumulate hydrophobic compounds.

### Summary

**Fig. 4:**
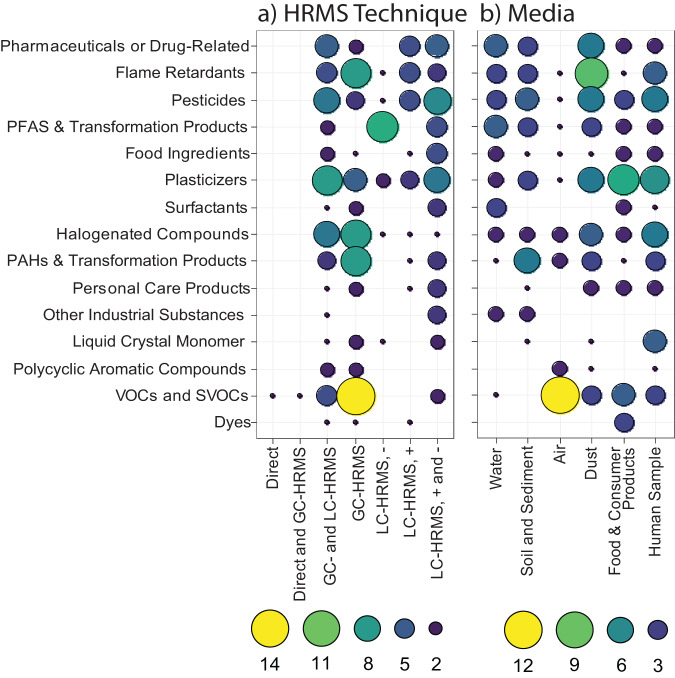
Chemical space detected in NTA by HRMS technique and environmental media. The chemicals detected using NTA by (**a**) HRMS technique used in the study and the (**b**) sample media studied. We examined the chemical classes that were detected in each of the 76 NTA studies reviewed herein, and the dot size and color represent the frequency the chemical class was reported in each study. In some studies, multiple chemical classes were reported and all chemical classes reported were considered in the frequency. For example, in El-Deen et al., wastewater was characterized using LC-HRMS and pharmaceuticals, flame retardants, pesticides, PFAS, plasticizers, and food ingredients were found.

A summary of the chemical space covered by each HRMS technique and each media discussed in this review are displayed in Fig. 4. The chemicals that were most frequently detected in each media were: PFAS and pharmaceuticals in water, pesticides and PAHs in soil and sediment, volatile and semi-volatile organic compounds in air, flame retardants in dust, plasticizers in consumer products, and plasticizers, pesticides, and halogenated compounds in human samples. Using GC-HRMS, VOCs and SVOCs, PAHs, halogenated compounds, and flame retardants were frequently detected in NTA workflows. Using LC-HRMS, PFAS were most frequently detected in negative ionization and pharmaceuticals, flame retardants, and pesticides were detected in positive ionization. In papers that used multiple methods (i.e., LC-HRMS in both positive and negative ionization or GC- and LC-HRMS), a wider range of compounds were detected in NTA.

## Gaps in current knowledge and technology

The primary goal of NTA for exposure studies is to characterize sources and types of understudied or unknown exposures and link them to human health. Ideally, for this purpose, NTA provides a broad chemical characterization of a sample (blood, soil, urine, etc.). NTA using HRMS in tandem with one or more separation techniques is the best technology for addressing this need as it is highly sensitive, comprehensive, and robust. However, NTA is not without limitations, including challenges in (1) the comprehensiveness of a single analytical technique, (2) ability to elucidate exact chemical structures, and (3) ability to accurately quantitate identified chemicals. These three limitations are discussed in more detail below and should be made clear to end users so that approaches are properly applied, and data is properly interpreted.

The first limitation is that no NTA technique is fully comprehensive; as such, multiple steps along the process of sample processing and preparation, data acquisition, and data-analysis will be selective to certain chemicals. For example, during sample extraction for LC only certain soluble chemicals will be recovered and separated for analysis, whereas in GC primarily certain volatile or semi-volatile molecules will be introduced into the mass spectrometer. Furthermore, certain solvents, extraction conditions, and other sample handling and processing procedures will lead to degradation, transformation, or losses of molecules of interest [89, 100]. For example, using a jet stream with heat to dry a sample can lead to loss of volatile or semi-volatile chemicals. Freeze thaw cycles, pulverizing, and bench top temperatures during extraction can lead to enzymatic degradation in biological samples, and certain solvents may have chemical reactions with compounds of interest with or without the presence of enzymes. Once introduced into the mass spectrometer, depending on ionization method, chemistries will determine which of the remaining chemicals are efficiently ionized and hence detectable. Furthermore, in-source fragmentation or other chemical or physical reaction in the mass spectrometer can lead to further losses of chemicals [101], and during data-processing artifacts, overlapping spectra, blank contamination, filter thresholds, and other effects can also lead to false negatives (missing molecules) [3]. Therefore, from an exposomics standpoint, multiple techniques may be needed which are complimentary (e.g., GC and LC-HRMS), and it must be accepted that not all chemicals will be captured.

The second limitation is that often exact molecular structure cannot be determined without a chemical standard (e.g., distinguishing between isomers with subtle structural differences including double bond position, branched versus linear chains, and stereoisomers) and unique standards do not exist for many compounds. For example, in GC-EI even if ionization occurs, often providing rich fragmentation evidence, the molecular ion often is not above the noise level, and hence positive identification is challenging without an alternative ionization technique, retention index match, or retention time match from a standard. In LC-ESI, the opposite often occurs, the precursor ion is observed at low abundance for trace environmental analysis, but the fragmentation spectra provides minimal to no fragments above the noise if the ion is selected for fragmentation at all, again necessitating a retention time match from a standard. Fragmentation spectra often provide the most structural information, but often these data provide only substructures (for example existence of a carbon fluorine chain and sulfonic acid head group for PFSAs) but not subtle isomeric differences (e.g., position of a double bond, branching, and chirality) [102, 103]. This subtle structural detail cannot always be ignored when assessing toxicity, as subtle chemical differences can influence toxicity dramatically [104]. Furthermore, if suspect screening libraries or other chemical databases do not contain the chemical of interest it is less likely to be determined, although in NTA new compounds never before reported can be discovered. Along these lines, many researchers employ in-house screening lists that are related to their specific area of interest, which could limit or bias the chemical profiles. As a result, moving towards a more general screening approach with more extensive libraries, such as the NORMAN database, would enable a more comprehensive analysis of the exposome. The incorporation of HRMS alongside the ever-growing library bank means compounds outside the scope of the original project can be targeted alongside specific compound classes of interest.

The third limitation is in quantitation, arguably the most challenging aspect of NTA. Instrument response often depends on chemical structure, and therefore ideally a response curve using internal standards is used for quantitation. For NTA, internal or external standards may not exist and therefore approaches predicted ionization efficiencies based on chemical structure and/or using surrogate standards with as similar chemical structure as possible can be used [11, 13, 47, 66]. While sophisticated approaches to obtain better quantitation are being developed, current predictions of concentrations (predicted upper confidence limits (protective values)) can be expected to be within 1 to 2-orders of magnitude of the true value [105–107]. While chromatography influences quantitation in both GC and LC, this issue is especially challenging for LC where ionization is structure specific, whereas in GC, ionization is not structure specific.

Errors associated with structural assignment and quantitation propagate into errors in assessment of exposure, toxicity, and risk, given that both structure and concentration are helpful for predicting toxicities. It is important to understand these limitations when reporting or interpreting NTA data so that risk is appropriately determined from chemical exposure. Therefore, efforts to properly report confidence in assigned chemical structures [61, 108], to calculate error in semi-quantitation using NTA [109, 110], and to verify results with known benchmarks [62], are essential. Provided that the assumptions and limitations are properly known, NTA data can be appropriately applied to various exposure scenarios providing insights that no other technique can.

## Implications and public health relevancy

The growing use of NTA in characterization of environmental matrices and human specimens has provided impactful benefits to public health. NTA can enable more comprehensive characterization of environmental exposures, allowing researchers to further understand their role in human disease. For example, the discovery of perfluoro-2-propoxypropanoic acid (also known as hexafluoropropylene oxide dimer acid, HFPO-DA, and the tradename “GenX”) in the Cape Fear River [27] using NTA led to monitoring of the drinking water in cities that used the river as a drinking water source, and ultimately resulted in monitoring the levels of GenX and related chemicals in the blood of residents in Wilmington, NC [109]. In medical devices, ISO 18562, a standardized NTA and SSA method, revealed that the polyester polyurethane foam in mechanical ventilator devices contained VOCs that were potentially toxic [111]. This discovery ultimately led to the recall of these devices.

In combination with other analysis, such as effect directed analysis (EDA), NTA can be implemented into chemical risk characterization and assessments to provide information regarding the toxicity of the detected analytes. Using a combination of sample preparation, sample fraction, and bioassays, EDA can be used to identify chemicals causing a particular type of adverse effect or response and indicate potential toxicity. EDA has been used to profile toxicity of both environmental media and human specimen. Several studies have paired NTA with EDA to evaluate soil toxicity using the aryl hydrocarbon receptor, estrogen receptor, and anti-androgenic receptor bioassays [40, 43, 45]. The assays revealed that historical and emerging PAHs, several PAH transformation products, musks, organophosphates, and steroids were agonists. In regard to human specimen, EDA has been used to profile the environmental chemicals found in amniotic fluid for estrogenic, androgenic, and dioxin-like activity [91]. Using these bioassays and freely available in vitro experimental data and in silico prediction tools, NTA identified several endocrine disrupting compounds, including diphenyl isophthalate and p,p’-ditolylamine, with limited to no information available on their use or production. Thus, pairing EDA with NTA can reveal which chemicals may be of toxicity concern and warrant further investigation.

NTA is an underutilized tool in intervention studies. In intervention studies, NTA can be particularly useful for identifying co-benefits or unintended consequences of interventions. During the COVID-19 pandemic, mobility restrictions caused a natural intervention on airborne environmental chemical levels and was examined with NTA/SSA [112]. The NTA of outdoor particulate matter revealed that compounds related to fossil fuel combustion (aromatic compounds with low degrees of unsaturation and long alkyl chains) decreased when mobility restrictions were in effect [112]. Along these lines, NTA could be used to develop and assess exposure mitigation strategies used for reducing environmental chemicals in other media (e.g., monitoring a water treatment before and after a technology is implemented).

## Conclusions

Due to the complexity of the exposome, choosing the appropriate approach (i.e., analytical platform, sample preparation and extraction technique, ionization mode, etc.) for NTA is challenging and understanding the chemical space for the intended analysis is critical. Based on the manuscripts reviewed herein, NTA studies performed to date have: (1) favored LC-HRMS for exposure characterization across various environmental media and human samples, (2) favored SSA over true NTA studies, likely due to the lack of open-source NTA software that covers multiple analytical platforms, and (3) not taken advantage of using multiple HRMS platforms to expand the detectable chemical space. Organizations, such as the BP4NTA working group (https://nontargetedanalysis.org), are working to address the need for harmonization and better reproducibility of NTA results. Continued investigation of exposure sources and exposures in epidemiological studies and integration of assessments using multiple analytical techniques are necessary to advance the scientific understanding of exposures to organic chemicals and their related health outcomes. As reviewed herein, much of the NTA research to date employs LC-HRMS, while GC-HRMS studies are less common. The use of multiple analytical techniques includes using both LC and GC based methods and the use of more than one ionization technique (e.g., positive and negative mode electrospray ionization, electron impact ionization, chemical ionization, atmospheric pressure chemical ionization, and atmospheric pressure photo ionization). To do so, experiments must be designed with chemical space in consideration through actions such as obtaining replicate samples for analysis on multiple platforms, using an extraction processing with solvents amenable to both techniques, and carefully considering the chemical contents of the sample media. Further, there is a need to develop open-source software for true NTA structure elucidation for multiple HRMS platforms. NTA can advance our scientific understanding of exposures by providing information about chemicals that may not have been suspected a priori. We encourage epidemiologists to work with analytical scientists to include NTA in their studies to more fully capture and understand exposure.

## Data Availability

Data available within the article or its supplementary materials.
